# TNM classification: clarification of number of regional lymph nodes for pN0

**DOI:** 10.1054/bjoc.2001.1996

**Published:** 2001-09


BJOC 01-1996 780 20/8/01 3:56 pm Page 780

*British Journal of Cancer* (2001) **85**(5), 780
© 2001 Cancer Research Campaign
doi: 10.1054/ bjoc.2001.1996, available online at http://www.idealibrary.com on IDEAL
http://www.bjcancer.com

Letters to the Editor

### TNM classification: clarification of number of regional lymph nodes for pN0

**Sir**,
In the January 2001 issue of the *British Journal of Cancer* Klein Kranenbarg et al (2001) evaluated the latest TNM classification of gastric carcinoma (Sobin and Wittekind, 1997) and found the N (regional lymph node) classification to be the most important prognostic variable and that the 1997 edition's N classification had higher prognostic value than the N in the previous edition (Hermanek and Sobin, 1987). Although we are pleased with these findings, a clarification is needed regarding the number of regional lymph nodes needed to classify a case as pN0.

The authors believed there was a 'requirement for the examination of at least 15 nodes to justify the N0 status.' Consequently, 'patients with fewer examined negative nodes are unclassifiable (NX).'

The 1997 TNM gastric carcinoma classification states (Sobin and Wittekind, 1997): 'pN0 Histological examination of a regional lymphadenectomy specimen will ordinarily include 15 or more lymph nodes.' (This applies only to the pathological assessment, pN0, and not to the clinical N0 status.)

This statement was not intended to be a requirement for pN0, but rather a guideline. In fact, the TNM Supplement (Hermanek et al, 1993) notes for all tumour sites: 'If the examined lymph nodes are negative, but the number ordinarily resected is not met, classify as pN0.'

We hope you will call this to the attention of your readers so that similar cases need not be excluded from analysis. We will make this guideline more explicit in the next edition of TNM.

Questions regarding TNM classification can be addressed to the TNM helpdesk at http://tnm.uicc.org.

**LH Sobin**
Chairman, TNM Committee
Division of Gastrointestinal Pathology
Armed Forces Institute of Pathology
Washington, DC 20306, USA

### REFERENCES

Hermanek P, Henson DE, Hutter RVP and Sobin LH (editors) (1993) *TNM Supplement 1993. A commentary on uniform use*. Springer: Berlin, Heidelberg, New York, p 49
Hermanek P and Sobin LH (editors) (1987) *TNM classification of malignant tumours*. 4th edition. Springer: Berlin, Heidelberg, New York,p 43–46
Klein Kranenbarg E, Hermans J, van Krieken JHJM and van de Velde CJH (2001) Evaluation of the 5th edition of the TNM classification for gastric cancer: improved prognostic value. *Br J Cancer* **84**: 64–71
Sobin LH and Wittekind Ch (editors) (1997) *TNM classification of malignant tumours*. 5th edition. Wiley: New York, p 59–62

### A reply

**Sir**,
We are glad that our interpretation of the number of 15 examined negative nodes was too rigorous and that the confusion has been solved. Many other authors (Fujii et al, 1998; Roder et al, 1998; Ichikura et al, 1999; Kato et al, 1999) had the same assumption and excluded patients where < 15 nodes were examined. We could at least prove that 15 nodes was too strict and that less nodes were also sufficient. The main finding remains that the new classification had a better prognostic value.

**E Klein Kranenbarg**
Department of Surgery
Leiden University Medical Center
PO Box 9600
2300 RC Leiden, The Netherlands

### REFERENCES

Fujii K, Isozaki H, Okajima K, Nomura E, Niki M, Sako S, Izumi N, Mabuchi H, Nishiguchi K and Tanigawa N (1999) Clinical evaluation of lymph node metastasis in gastric cancer defined by the fifth edition of the TNM classification in comparison with the Japanese system. *Br J Surg* **86**: 685–689
Ichikura T, Tomimatsu S, Uefuji K, Kimura M, Uchida T, Morita D and Mochizuki H (1999) Evaluation of the New American Joint Committee on Cancer/International Union against cancer classification of lymph node metastasis from gastric carcinoma in comparison with the Japanese classification. *Cancer* **86**: 553–558
Kato M, Saji S, Kawaguchi Y, Kunieda K, Sugiyama Y, Takagi Y, Hiraoka T, Kumazawa I and Adachi T (1999) A comparison of the prognostic significance between the number of metastatic lymph nodes and nodal stage in gastric carcinoma. *Hepatogastroenterology* **46**: 3281–3286
Roder JD, Bottcher K, Busch R, Wittekind C, Hermanek P and Siewert JR (1998) Classification of regional lymph node metastasis from gastric carcinoma. German Gastric Cancer Study Group. *Cancer* **82**: 621–631

